# Factors Contributing to Psychoactive Substance Use Among Nursing Students

**DOI:** 10.1192/j.eurpsy.2026.11851

**Published:** 2026-07-31

**Authors:** J. Ben Khelifa, F. Chelly, N. Gannoun, C. Sridi, N. Belhadj, R. Nakhli, A. Fki, I. Kacem, M. Maoua

**Affiliations:** 1 Clinical Psychology Unit, Occupational Medicine Department; 2 Occupational Medicine Department, Sahloul University Hospital; 3 Faculty of Medicine of Sousse, University of Sousse; 4 Sousse, Research Laboratory (LR19SP03); 5 Occupational Medicine Department, Farhat Hached University Hospital, Sousse, Tunisia

## Abstract

**Introduction:**

The rising prevalence of psychoactive substance (PAS) use among nursing students is a growing concern that poses significant public health challenges, reflecting a complex interplay of individual, social, and environmental influences.

**Objectives:**

This study aims to identify the key factors contributing to psychoactive substance use among nursing students.

**Methods:**

We conducted a descriptive cross-sectional survey with 300 students at the Private Higher Institute of Nursing Sciences in Sousse (ISIS) during November and December 2024. Data were collected using a self-administered questionnaire.

**Results:**

The etiopathogenic factors determining the use of drugs among students may be individual, manifested as personality disorders according to 53.66% of respondents, or familial, as reported by 50.33% of students. Among the surveyed students, 37.9% have been using drugs for over a year, while 25.7% have been using them for less than a year. However, 37% have never used drugs. Social pressure and peer influence also represented significant reasons for use, reported by 69.31% of respondents. Among drug users, 49.73% were seeking well-being, and 53.96% desired to experience euphoria. Regarding academic challenges, 26% of students reported experiencing difficulties with their university studies. The primary cause identified was excessive workload, mentioned by 32.05% of the respondents. Poor time-management skills were reported by 19.23% as another contributing factor. Furthermore, 18% of students perceived time management as having a moderate impact, while 16.32% considered it highly significant, with 10.66% indicating ‘a lot’ and 5.66% indicating ‘extremely’ influential.

**Conclusions:**

This study highlights the multifactorial nature of psychoactive substance use among nursing students, influenced by both psychosocial and academic stressors. Further research should explore effective support systems in nursing education and assess the impact of mental health programs on reducing substance use.

**Disclosure of Interest:**

None Declared

